# From facial expressions to algorithms: a narrative review of animal pain recognition technologies

**DOI:** 10.3389/fvets.2024.1436795

**Published:** 2024-07-17

**Authors:** Ludovica Chiavaccini, Anjali Gupta, Guido Chiavaccini

**Affiliations:** ^1^Department of Comparative, Diagnostic, and Population Medicine, College of Veterinary Medicine, University of Florida, Gainesville, FL, United States; ^2^Independent Researcher, Livorno, Italy

**Keywords:** AnimalFACS, computer vision, Convolutional Neural Networks (CNNs), deep learning, facial expressions, machine learning, pain recognition

## Abstract

Facial expressions are essential for communication and emotional expression across species. Despite the improvements brought by tools like the Horse Grimace Scale (HGS) in pain recognition in horses, their reliance on human identification of characteristic traits presents drawbacks such as subjectivity, training requirements, costs, and potential bias. Despite these challenges, the development of facial expression pain scales for animals has been making strides. To address these limitations, Automated Pain Recognition (APR) powered by Artificial Intelligence (AI) offers a promising advancement. Notably, computer vision and machine learning have revolutionized our approach to identifying and addressing pain in non-verbal patients, including animals, with profound implications for both veterinary medicine and animal welfare. By leveraging the capabilities of AI algorithms, we can construct sophisticated models capable of analyzing diverse data inputs, encompassing not only facial expressions but also body language, vocalizations, and physiological signals, to provide precise and objective evaluations of an animal's pain levels. While the advancement of APR holds great promise for improving animal welfare by enabling better pain management, it also brings forth the need to overcome data limitations, ensure ethical practices, and develop robust ground truth measures. This narrative review aimed to provide a comprehensive overview, tracing the journey from the initial application of facial expression recognition for the development of pain scales in animals to the recent application, evolution, and limitations of APR, thereby contributing to understanding this rapidly evolving field.

## 1 Introduction

In animals and humans, facial expressions play a crucial role as a primary non-verbal method for managing peer interactions and conveying information about emotional states (1). Scientific interest in facial expressions was initiated in the 1860s by Duchenne de Boulogne. However, it is in the last two decades that the utilization of facial expressions for understanding emotional conditions, such as pain, has expanded in both humans and non-human species (2). Notably, it was demonstrated that facial expressions of pain show consistency across ages, genders, cognitive states (e.g., non-communicative patients), and different types of pain and may correlate with self-report of pain in humans (3, 4). Analyzing facial expressions and body language in animals poses unique challenges absent in human medicine, like data collection, establishing ground truth—that is, determining whether or not the animal is experiencing pain or distress, and navigating the vast array of morphological differences, shapes, and colors present within and across animal species (5, 6). Various scales for interpreting facial expressions in animals have been created in the past decade. The Mouse Grimace Scale (MGS) was the first facial grimace scale for animal pain assessment, developed from studies on emotional contagion in mice, and led to the creation of similar scales for other species, such as the Rat Grimace Scale (RGS) (7, 8). These scales, now developed for 11 species, have been used in various pain models, including surgical procedures and husbandry practices. Despite their usefulness, limitations include the fact that most of these pain scales were developed based on a restricted number of action units (AUs) retrieved from picture-based recognition patterns, as described in more details later.

Computational tools, especially those based on computer vision (CV), provide an attractive alternative. Automated Pain Recognition (APR) is an innovative technology that utilizes image sensors and pain algorithms that employ Artificial Intelligence (AI) techniques to recognize pain in individuals (9, 10). These systems are based on machine learning (ML) techniques to recognize and classify facial expressions associated with pain (11). Machine learning consists of training an algorithm to discern various categories or events (classes). Subsequently, this trained algorithm is utilized to identify categories or events within a new or unknown data set. The application of AI optimized the research on classification algorithms of ML, increasing recognition rates, computing speed and preventing system crashes.

Machine learning and AI can radically change how we recognize and treat pain in non-verbal patients, including animals, with an immense impact on veterinary medicine and animal welfare. By harnessing the power of ML algorithms, we can create sophisticated models that analyze various data inputs, not only facial expressions but also body posture and gesture (12), vocalizations (13), and physiological parameters, to accurately and objectively assess an animal's pain level. This approach will enhance our ability to provide timely and effective pain management, and it will be pivotal in minimizing suffering and improving the overall quality of life for animals under our care.

Therefore, this narrative review aims to focus on the impact of automation in the recognition of animal somatosensory emotions like pain and to provide an update on APR methodologies tested in the veterinary medical field, as well as their differences, advantages, and limitations to date.

## 2 Facial expression-based (grimace) scales for animal pain assessment

A grimace pain scale assesses animals' pain by evaluating changes in their facial expressions. It is developed through systematic observation and analysis of facial expressions exhibited by animals in response to pain-inducing stimuli. Researchers identify specific facial features associated with pain and create a coding system to quantify these responses objectively. The scale then undergoes validation to establish its reliability and sensitivity.

The MGS was the pioneering facial grimace scale for pain assessment developed for animals, emerging from investigations exploring the possibility of emotional contagion in mice (14). These studies exposed the capacity of mice to discern pain in their counterparts through subtle changes in body language and facial expressions after they were injected intraperitoneally with 0.9% acetic acid (7). Within a short span, the RGS followed suit, its inception marked by experiments conducted on appendicular inflammatory models and a laparotomy model (8, 15). Demonstrating features mirroring those of the MGS—such as orbital tightening, ear changes, and whisker alterations—the RGS exhibited comparable reliability and accuracy. Moreover, it showcased sensitivity to morphine and the ability to quantify pain stemming from inflammatory sources (8). Since their development, rodent grimace scales have been tested in several preclinical pain models, including post-laparotomy (16), post-vasectomy (17), post-thoracotomy (18). Following the initial publications, there has been a swift expansion in both the conversation and application of grimace scales. Grimace scales have been developed for 11 distinct species, including rodents, lagomorph (19), feline (20, 21), equine (22, 23), bovine (24), swine (25, 26), ovine (27, 28), ferrets (29), harbor seals (30), and donkeys (31, 32). As castration is considered one of the most common surgical procedures practiced by veterinarians, it is not surprising that several of these models were based on difference in behavior and posture before and after castration (22, 24, 26, 31, 33–35). However, other husbandry procedures have been used, like tail docking, ear-tagging and microchipping (25, 28, 30). A complete overview of the facial grimace scales developed to date and the painful stimulus used has been reported in Table 1.

**Table 1:** Overview of facial expression-based pain scales developed to date.

**References**	**Species**	**Number of animals**	**Pain stimulus**	**Inputs**	**Image evaluation**	**Validation**
Yamada et al. (24)	Bovine	45	Castration	Still images (unspecified number)	Blind observer	No
Orth et al. (31)	Donkey	9	Castration	54 still images	12 observers different experience	No
van Dierendonck et al. (32)		264	Spontaneous pain	Direct observation	Six veterinary graduate students	No
Dalla Costa et al. (22)	Equine	46	Castration	126 still images	Blind experienced observer	No
Gleerup et al. (23)		6	Tourniquet application and capsaicin injection	36 still images	Professional scientific illustrator	No
van Loon and Van Dierendonck (36)		50	Spontaneous colic	Direct observation	Four unblind students	No
VanDierendonck and van Loon (37)		46	Spontaneous colic	Direct observation	Unspecified	No
Dalla Costa et al. (38)		10	Spontaneous laminitis	40 still images and videos	Four blinded observers	No
Holden et al. (20)	Feline	87	Spontaneous pain	16 still images	68 observers with different experience	No
Evangelista et al. (21)		55	Spontaneous pain	110 still images	Four observers different experience	Yes
Reijgwart et al. (29)	Ferret	19	Laparotomy	114 still images	11 observers different experience	No
MacRae et al. (30)	Harbor seal	47	Tagging and microchipping	98 clips	Two observers	No
Langford et al. (7)	Mouse	8–20 per assay	0.9% acetic acid abdominal constriction test and others	64 still images	Seven blinded graduate and undergraduate students	Partially
McLennan et al. (39)	Ovine	73	Spontaneous foot root and mastitis	60 still images	Six blinded observers	Partially
Häger et al. (27)		14	Unilateral osteotomy	66 still images	Six observers various experience	No
Guesgen et al. (28)	(lambs 5–6 weeks old)	7	Tail docking	56 still images	Five observers various experience	No
Keating et al. (19)	Rabbit	8	Ear tattooing	64 still images	10 observers various experience	Partially
Sotocinal et al. (8)	Rat	6–8	Various	104 still images	Five blinded graduate students	No
Di Giminiani et al. (25)	Swine (piglets 3 and 4-day old)	23	Tail docking and castration	94 still pictures (combined)	Unspecified number of experienced observers	No
Viscardi et al. (26)	(piglets 5-day old)	19	Castration	627 still images	One blinded experienced observer	No
Viscardi and Turner (35)	(piglets 5-day old)	60	Castration	511 still images	Four blinded observers	No
Viscardi and Turner (34)	(piglets 5-day old)	120	Castration	1,156 still images	Eight blinded observers	No

Studies have been categorized by species, number of animals used to create the scale, type of pain stimulus, kind of data input, facial information processing, and whether the scale underwent full validation.

These studies collectively share several common limitations. Primarily, a significant inconsistency exists in developing species-specific ethograms associated with pain. An ethogram is a descriptive inventory or catalog of all behaviors or actions exhibited by a particular species or group of animals under specific conditions. But many of these investigations were conducted before establishing a formal codification system for facial expressions in the relevant species, such as the Facial Action Coding System (FACS), which will be elaborated upon in the subsequent paragraph. A wide range of pain models has been employed across these studies, including experimental models (7, 8, 23, 29), clinical or husbandry procedures (24, 26, 31, 34, 35, 40) and observations of spontaneous pain (20, 21, 36–39). Notably, it has been demonstrated that the duration of the noxious stimulus affects the facial expression of pain (14). Langford et al. (7) showed that noxious stimuli lasting between 10 min and 4 h were most likely to elicit a “pain face.” Consequently, this would render most transient pain models (30) and chronic pain models (39, 41) inadequate for facial pain detection. Interesting ear notching did not evocate grimace in mice (42) but it did in rabbits (19). Furthermore, potential overlap between pain and other states (sleep, grooming, and illness) has been observed (43, 44). In many cases, animals were assessed both before and after procedures requiring general anesthesia (8, 22, 27, 29, 31). However, studies have shown that the facial expression of pain can remain altered for several hours after inhalant anesthesia in both experimental mice and rats (45, 46) and in horses (47). This effect likely holds for other animal species.

The collection of images for facial expression scoring lacked consistency across studies. Despite trained personnel being capable of regularly recording and evaluating animal pain intensity in clinical settings, continuous annotation still needs to be attainable (48). Many studies relied on static images, often arbitrarily extracted from videos of varying durations, or real-time scoring, with manual annotation performed by human researchers. This approach introduced the risk of bias and subjective judgment. Furthermore, researchers emphasized the necessity of using high-definition video cameras or still cameras to ensure optimal image quality (7, 22, 25, 27, 43) but to avoid the use of bright light or camera flashes (20). The development of the RGS coincided with the introduction of the Rodent Face Finder^®^ free software, designed to streamline the conversion of videos into scorable photographs by capturing frames with optimal optical quality and head positioning (8). Similar approaches have been developed also for horses (49). Typically, images were then pre-processed, with cropping around the head and removal of the background being common practices. However, the impact of background on image interpretation remains untested (43). Subsequently, these still images were presented to blind observers with varying levels of experience to assess inter- and intra-rater variabilities. Notably, observer experience significantly impacted the ability to discern facial features (7, 28, 31, 39). Given animals' inability to verbalize pain and the variability in employed pain models, researchers have typically identified facial changes occurring in more than 25–50% of animals following a painful stimulus as indicative of pain (22, 24, 29). Alternatively, they have relied on the coding of pain AUs recognized by experts in human facial pain expression (7, 23). But it is known that human observers often categorize facial expressions based on emotion, which can influence the process of comparing expressions across different species (50).

Construct validity of the pain scale is typically assessed by comparing the scores of animals experiencing pain vs. those undergoing sham procedures and by reassessing the painful animal before and after treatment. However, in the existing literature these comparisons were often omitted due to ethical concerns with performing invasive veterinary procedures without analgesia (22). Dalla Costa et al. (22) found no differences in the Horse Grimace Scale (HGS) among horses undergoing castration under general anesthesia, regardless of receiving one or two doses of flunixin meglumine. Similarly, there were no differences in the Piglet Grimace Scale (PGS) scores between piglets castrated with and without receiving meloxicam (34) or piglets receiving buprenorphine injections whether undergoing castration or not (35). Even when PGS was refined through 3D landmark geometric morphometrics, neither the PGS nor 3D landmark-based geometric morphometrics were able to identify facial indicators of pain in piglets undergoing castration (51). These findings raise questions about the potential confounding effects of drugs and the reliability of the scale in assessing post-castration pain. While this is not substantiated by the current literature, it is also possible that expressions may not always be an accurate indicator of pain in animals or researchers did not identify the pain ethogram for the species yet.

While animals cannot communicate their pain perception directly, the criterion validity of a pain scale can be assessed by testing it against a gold standard. However, this validation method was rarely conducted in previous studies (21, 27, 39, 41). A pain scale's internal consistency measures its components' coherence. In pain assessment, a scale demonstrates internal consistency if it consistently yields similar scores for the same aspect of pain across its various items or questions. This ensures that all items reliably measure the exact dimension of pain. Internal consistency is typically assessed using statistical methods like Cronbach's α coefficient, with higher values indicating more robust agreement among scale items and more reliable pain measurement. However, internal consistency has been reported only for the Feline Grimace Scale (21). Inter- and intra-rater reliability assess the agreement among different raters (inter-rater reliability) or the same rater over multiple assessments (intra-rater reliability) when using the scale to evaluate pain. Inter-rater reliability ensures consistent results regardless of who administers the scale, ensuring validity and generalizability across different observers. Intra-rater reliability confirms the stability and consistency of the scale's measurements over time, indicating that a rater's assessments are not influenced by variability or bias. The Intraclass Correlation Coefficient (ICC) is widely used to measure reliability, with values <0.50 indicating poor agreement, between 0.50 and 0.75 indicating moderate agreement, between 0.75 and 0.90 indicating good agreement, and above 0.90 indicating excellent agreement (52). Inter-rater ICC values for current facial expression pain scales ranged between 0.57 (26) and 0.92 (27), while intra-rater ICC ranged between 0.64 (24) and 0.90 (21), with considerable variability across facial features. But presenting good rater agreement on a given behavior does not mean that the behavior actually measures a given emotion. Another significant limitation of existing facial pain scales is the need for a cutoff value for treatment determination. van Loon and Van Dierendonck (36) reported that the EQUUS FAP had sensitivity and sensibility for distinguishing colic from no-colic of 87.5 and 88% using a cut-off value of 4 in a scale 0–18, but only of 30 and 64.3% for distinguishing surgical and medical colic with a cut-off at 6. Häger et al. (27) and McLennan and Mahmoud (53) both reported a discrimination accuracy below 70% using two different facial pain scales developed for sheep, denouncing a high number of false positive results and highlighting the need for further refinement and standardization in this area.

## 3 Facial Action Coding System

The gold standard for objectively assessing changes in facial expressions in human emotion research is the FACS, first published almost half a century ago (54). FACS is a comprehensive, anatomically based system that taxonomizes all visible human facial movements (55, 56). In FACS, the authors assign numbers to refer to the appearance changes associated with 33 facial muscle contractions to each specific facial movement, termed AUs. Each AU is linked to mimetic muscles innervated by the facial nerve and characterized by corresponding changes in facial appearance. Additionally, the system introduced 25 more general head/eye movements termed Action Descriptors (AD), representing broader movements from non-mimetic muscles, which could impact AU identification. Recognizing the interplay between AUs and ADs is emphasized, as their concurrent presence could modify the visual expression of individual movements. The FACS manual offers guidelines for scoring these AUs, supported by a collection of photographs and illustrations for reference. The FACS system revolutionized human research based on facial expression interpretation, finding extensive application in psychology, sociology, and communication. It enabled the objective and systematic recognition of individual facial movements based on facial anatomy and steered the field away from subjective interpretations of visual displays known for their unreliability. Following the FACS approach, researchers have developed the same system for non-human primates, including orangutans [*Pongo* spp: OrangFACS (55)], chimpanzees chimpanzees [*Pan troglodytes*: ChimpFACS (56)], rhesus macaques [*Macaca mulatta*: MaqFACS (57, 58)], gibbons [Hylobatids, GibbonFACS (59)], marmosets (60), and domesticated mammals such as horses [*Equus caballlus*: EquiFACS (61, 62)], dogs (*Canis familiaris*: DogFACS) (63), and cats (*Felis catus*: CatFACS) (64).

Developing species-specific AnimalFACS involved identifying and documenting every potential facial movement of the species based on observable changes in appearance, consistent with the FACS terminology. Subsequently, the muscular foundation of each movement was confirmed through rigorous anatomical studies (56, 61, 63). This extensive work has interestingly unveiled phylogenetic similarities across species, with those already analyzed for FACS demonstrating a shared muscular foundation of at least 47% of their facial muscles (65). While species may share similar anatomical structures, this correspondence does not invariably translate into analogous facial movements. Specific muscles may be implicated in multiple AUs, while others may exhibit infrequent use, complicating the relationship between anatomy and expression (65). For a more detailed description of all the AUs identified in the different species, the reader is referred to Waller et al. (65). But, while FACS is generally considered reliable for gauging human perception due to the presumed alignment between facial expression production and interpretation, its applicability to non-human animals may be less precise, as third party evaluation is always required. Therefore, it's vital to approach its application cautiously and gather empirical data to ascertain how animals respond to stimuli.

Despite the growing interest in facial expression analysis for evaluating pain and emotion, only a few animal studies applied AnimalFACS. Among small animal species, the FACS system has been scarcely used. In dogs and cats, FACS has been used more commonly for emotion interpretation than specifically for pain determination (66–68). In one study, 932 images from 29 cats undergoing ovariohysterectomy were extracted and manually annotated using 48 landmarks selected according to CatFACS criteria (69). A significant relationship was found between pain-associated Principal Components, which capture facial shape variations, and the UNESP-Botucatu Multidimensional Composite Pain Scale tool (69). However, an intrinsic bias of the study was that the first postoperative assessment, prior to administration of analgesia, was recorded between 30 min and 1 h after general anesthesia, and the role of general anesthesia on facial expression cannot be excluded as it has been previously discussed. A groundbreaking methodology for investigating the facial expressions of ridden horses, known as Facial Expressions of Ridden Horses (FEReq) (70, 71), was developed by integrating species-specific ethograms from previous studies (22, 23) with components of the EquiFACS codification system (61). This ethogram represented a pioneering effort in characterizing changes in facial expressions among ridden horses, demonstrating reasonable consistency across diverse professional backgrounds post-adaptation and training. Although initially limited to analyzing still photographs capturing singular moments, the ethogram was subsequently enhanced with additional markers for assessing general body language and behavior in ridden horses (72). Despite no observed correlation between this improved Ridden Horse Pain Ethogram (RHpE) score and maximum lameness grade before diagnostic anesthesia (Spearman's rho = 0.09, *P* = 0.262) (73), the scale has proven effective in detecting musculoskeletal pain in competitively ridden horses (74, 75). These studies uncovered variations in consistency across horse facial features, particularly noting the eye and muzzle as displaying the least reliability. This stands in contrast to findings by Rashid et al. (62), who repurposed data from Gleerup et al. (23) to employ EquiFACS in describing facial features in pain-related videos. The group suggested that *inner brow raiser* (AU101), *half blink* (AU47), *chin raiser* (AU17), *ear rotator* (EAD104), *eye white increase* (AD1), and *nostril dilator* (AD38) were frequently linked with pain. Moreover, these findings were echoed by a recent study by Ask et al. (76), investigating pain indicators in horses with experimentally induced orthopedic pain. Employing the Composite Orthopedic Pain Scale (77) as the gold standard, the group identified numerous lip and eye-related AUs and ADs as robust predictors of pain. Noteworthy indicators included frequency and duration of *eye closure* (AU143), duration of *blink* (AU145), *upper lid raiser* (AU5), duration of *lower jaw thrust* (AD29), frequency and duration of *lower lip relax* (AD160), frequency of lower *lip depressor* (AU16), frequency of *upper lip raiser* (AU10), frequency and duration of AU17, duration of *lip presser* (AU24), frequency and duration of AD38, and frequency and duration of *lips part* (AU25), among others. Additionally, AU16, AU25, AU47, single ear forward (SEAD101), and EAD104 co-occurred more frequently in horses experiencing orthopedic pain. The study by Rashid et al. (62) also noted an interesting discrepancy in pain detection rates. In still images or video segments lasting 0.04 s, the likelihood of detecting more than three pain AUs was extremely low, contrasting with higher detection rates with a 5 s observation window. This may be explained by the fact that 75% of pain-related AUs in horses lasts between 0.3 and 0.7 s (76). This finding underscores the potential value of using video footage over randomly selected images for pain assessment. However, it's essential to acknowledge limitations in these studies, such as the small number of experimental horses used to build the models and the presumption of pain based solely on evaluations by clinically experienced observers, potentially overlooking influences of stress, tiredness and malaise (44).

One of the limitations of AnimalFACS consist in the limited availability across species and the reliance on manual annotation, necessitating rigorous human training to ensure acceptable inter-rater reliability (78, 79). Debates arose regarding distinctive individual differences, encompassing variations in muscle presence, size, symmetry, disparities in adipose tissue distribution, and even inherent facial asymmetry (65, 80). Notably, present studies using AnimalFACS are limited to quantifying the number of AUs, their combinations, and their temporal duration within a confined observation period (62, 72). However, this approach falls short of capturing the intricate complexity of facial movements. Another fundamental limitation of FACS-based systems is their failure to account for the dynamic shifts in movement or posture that often accompany and enrich facial expressions. So, some studies have assessed behavioral indicators such as changes in consumption behaviors (time activity budgets for eating, drinking, or sleeping, etc.) (81–83); anticipatory behaviors (84), affiliative behavior (85), agonistic behaviors, and displacement behaviors, amongst others (86).

## 4 Automated pain recognition

Automated Pain Recognition is a cutting-edge technology aiming to develop objective, standardized, and generalizable instruments for pain assessment in numerous clinical contexts. This innovative approach has the potential to significantly enhance the pain recognition process. Automated Pain Recognition leverages image sensors and pain algorithms, powered by AI techniques, to identify pain in individuals (9, 10). AI, a field encompassing a broad range of symbolic and statistical approaches to learning and reasoning, mimics various aspects of human brain function. Data-driven AI models, such as those used in APR, can overcome the limitations of subjective pain evaluation. Machine learning, CV, fuzzy logic (FL), and natural language processing (NLP) are commonly considered subsets of AI. However, with technological advancements and interdisciplinary research, the boundaries between these subsets often blur. Machine learning, a branch of AI, enables systems to learn and improve their performance through experience without explicit programming. It involves training a computer model on a dataset, allowing it to make predictions or decisions independently. Automated Pain Recognition research has focused on discerning pain and pain intensity within clinical settings (87) and assessing responses to quantitative sensory testing in preclinical research (88, 89). The following paragraphs will briefly outline and summarize the steps involved in APR.

### 4.1 Data collection

The initial step toward implementing APR involves data collection, a significant challenge in the veterinary field due to the scarcity of available datasets (90). Animals exhibit considerable variability even within the same species, influenced by factors such as breed, age, sex, and neuter status, that may affect the morphometry of the face, especially in adult males (91). These variables can impact the pain-related facial information extracted from images (6, 92, 93). This variability, however, can enhance the learning process of deep learning (DL) models. Exposure to diverse examples and scenarios allows models trained on a broad spectrum of data to generalize well to unseen examples, improving performance in real-world applications. Additionally, variability aids in acquiring robust features applicable across different contexts. With the availability of high-definition cameras and the relatively low demand for image or video quality in CV, recording has become less problematic compared to the past (49). Studies suggest that resolutions of 224 × 224 pixels and frame rates of 25 FPS are sufficient for processing images and videos in modern CV systems (49). Multicamera setups are ideal, especially for coding both sides of the face, as required in laterality studies or to avoid invisibility. Different animal species pose unique challenges. Laboratory animals are usually confined to a limited environment, allowing more control over data acquisition and video recording quality (89, 94, 95). Horses can be manually restrained or confined in a stall (96). Data acquisition for farm animals often occurs in open spaces or farms with uncontrolled light conditions (53, 97).

### 4.2 Data labeling

The absence of verbal communication in veterinary APR introduces a unique challenge in establishing a ground truth label of pain or emotional state. Unlike human medicine, where self-reporting of pain is feasible, veterinary APR requires third-party assessment of the pain status, preferably utilizing a validated pain scale, but commonly not (Table 2). This has led to the categorization of pain labeling methods in animal APR into behavior-based or stimulus-based annotations (90). The former relies solely on observed behaviors and is typically assessed by human experts (5, 6, 97, 99, 104–106). In contrast, the latter determines the ground truth based on whether the data were recorded during an ongoing stimulus or not (5, 10, 49, 76, 94–96, 99, 100, 107–109). Stimulus-based annotations enable recording the same animal under pain and no pain conditions and offer a potential solution to the challenge of variability in pain perceptions across individuals (110). Therefore, CV and ML methods must acknowledge the inherent bias in their algorithms until a definitive marker for pain is identified.

**Table 2:** Overview of datasets featuring facial expressions of pain for automated animal pain assessment to date.

**References**	**Species**	**Pain stimulus**	**Data**	**Input**	**Part/holistic**	**Approach**	**Annotation**
Broomé et al. (98)	Equine	Tourniquet application and capsaicin injection	60 video from six healthy horses	Videos	Holistic	Learned	Binary
Broomé et al. (96)		Tourniquet application, capsaicin injection and lipopolysaccharides induced lameness	Dataset from (98) and 90 videos from eight healthy horses	Videos and frames	Holistic	Learned	Binary
Hummel et al. (99)		Induced and unknown	1,854 images of horse heads and 531 images of donkey heads	Frames	Parts-based	Hand-crafted and Learned	HGS and EQUUS-FAP
Lencioni et al. (10)		Castration	3,000 frames from seven healthy horses	Frames	Parts-based	Learned	HGS
Pessanha et al. (5)		Induced or unknown	1,854 images of horse heads	Frames	Parts-based	Hand-crafted and Learned	Adapted from EQUUS-FAP
Feighelstein et al. (100)	Feline	Ovariohysterectomy	464 images from 26 cats	Frames	Holistic	Hand-crafted and Learned	Binary
Feighelstein et al. (6)		Unknown	84 client-owned cats	Frames	Holistic	Hand-crafted and Learned	Binary (based on CMPS-feline)
Martvel et al. (101)		Ovariohysterectomy	54 videos from 27 cats + 72 videos from client-owned cats	Frames	Holistic	Hand-crafted	CMPS-feline
Feighelstein et al. (102)	Lagomorphs	Orthopedic surgery	48 videos from 28 rabbits	Frames	Holistic	Learned	Binary
Tuttle et al. (94)	Murine	Laparotomy	5,771 frames	Frames	Holistic	Learned	Binary
Andresen et al. (95)		Castration	18,273 frames	Frames	Holistic	Learned	Binary
Lu et al. (103)	Ovine	Unknown	480 frames	Frames	Holistic	Hand-crafted	SPFS
Mahmoud et al. (104)		Mastitis and pregnancy toxemia	86 frames	Frames	Parts-based	Hand-crafted	SPFS
Noor et al. (97)		Unknown	2,350 frames	Frames	Holistic	Learned	Binary
Pessanha et al. (105)		Mastitis and pregnancy toxemia	86 frames	Frames	Parts-based	Hand-crafted	SPFS

Studies have been categorized by species, kind of pain stimulus, type of data input, facial information processing, whether the approach was hand-crafted or learned and the kind of pain annotation.

CMPS-feline, Glasgow Feline Composite Measure Pain Scale; EQUUS-FAP, Equine Utrecht University scale for facial pain assessment; HGS, Horse Grimace Scale; SPFS, Sheep Pain Facial Expression Scale.

### 4.3 Data analysis

Computer vision-based methods operate using data in the form of images or image sequences (videos). This suggests that the system can utilize single frames, aggregate frames (10) or incorporate spatiotemporal representations to account for temporality (94, 98, 105). Utilizing single frames offers greater control and facilitates explainability, although it may result in information loss. Researchers demonstrated that the likelihood of observing more than three pain AUs was negligible in still images extracted from videos of horses undergoing moderate experimental nociceptive stimulus (62). On the other hand, based on Martvel et al. (101), different frame extraction rates may affect the accuracy of the results. Preliminary results in mice (94), horses (98), sheep (105), and cats (101) suggest that extracting spatiotemporal patterns from video data may increase the performance of the model. However, working with videos rather than single-frame input requires substantial computational resources.

The data processing pipeline is developed after the images are collected and the input is either images or videos. The output is typically pain classification, which can be binary pain/no pain or multi-class degree assessment. Often, outputs based on grimace pain scale taxonomy encompass at least three scales [pain not present (0), pain moderately present (1), or pain present (2)] (10, 103). The pipeline can encompass multiple steps and may analyze the entire body or face or focus on specific parts. These two approaches, differing in processing facial information, have been defined as parts-based and holistic methods. For instance, Hummel et al. (99) cropped the equine face based on several Regions of Interest (ROIs); the eyes, ears, nostrils, and mouth, respectively, and analyzed them with HOG (Histogram of Oriented Gradients), Local Binary Pattern (LBP), Scale Invariant Feature Transform (SIFT), and DL approach using VGG-16 Convolutional Neural Network (CNN). Similarly, Lencioni et al. (10) employed a parts-based approach in annotating 3,000 images from seven horses of similar breeds and ages undergoing castration. They utilized the HGS (22), where the six parameters were grouped into three different facial parts: ears, eyes, and muzzle. Subsequently, three pain classifier models based on CNN architecture were developed. The outputs of these models were then fused using a fully connected network for an overall pain classification. Recent research employing explainable AI methods to investigate different regions of cat faces suggested that features related to the ears may be the least important (111). In contrast, those associated with the mouth movement were considered the most crucial (6, 49). Similarly, Lu et al. (103) have developed a multilevel pipeline to assess pain in sheep, utilizing the Sheep Facial Expression Pain Scale (39). The authors divided the sheep's face into regions, including eyes, ears, and nose, with further subdivision of the ears into left and right. Symmetric features such as eyes and ears were scored separately and then averaged, while scores for all three facial features (ears, eyes, nose) were averaged again to derive the overall pain score. The task of automatically identifying and localizing specific points or features on an animal's face, such as the eyes, nose, mouth corners, etc., known in CV as recognition of *key facial points*, poses the initial challenge due to limited datasets in animals (95). Researchers have proposed adapting animal training data to a pre-trained human *key point* detector to address this issue. The approach involved morphing animal faces into human faces and fine-tuning a CNN developed for human *key point* recognition. Surprisingly, this approach has demonstrated promising performance in both equine and ovine faces (112).

### 4.4 Hand-crafted vs. deep learning

Automated Pain Recognition identifies, understands, and enhances image pain features. Two main approaches have been used for feature extraction.

#### 4.4.1 Hand-crafted features extraction

Before the advent of DL, classical ML relied on hand-crafted features (90). The process involves extracting characteristics from the data using previous knowledge to capture pain-related patterns with facial or bodily landmarks, grimace scale elements, or pose representations. For example, Blumrosen et al. (113) studied four fundamental facial movements to recognize facial actions in macaques: neutral expression, lip smacking, chewing, and random mouth opening. They used unsupervised learning, which does not require manually labeling or annotating the data. In their approach, they utilized eigenfaces to extract features from facial images. Eigenfaces use a mathematical method called Principal Component Analysis (PCA) to capture the statistical patterns present in facial images. Another standard method is the landmark-based (LM-based) approach, which identifies pain-related AUs through manual annotation (7, 10, 94, 103). It provides a mathematical representation of previous findings by human experts concerning certain facial expressions. The system requires preliminary efforts to detect and locate the animal face in an image or video clip and to detect individual AUs. Face detection and alignment are achieved by detecting *key facial points*, which are then transformed into multi-region vectors and fed to a multi-layer perceptron neural network (MLP). For example, Andersen et al. (49) trained individual classifiers to detect 31 AUs, including ADs and ear EADs, in 20,000 EquiFACS-labeled short video clips after cropping the images around a pre-defined ROI to help the classifier focus on the correct anatomical region. But the model did not work for the ear action descriptors. The authors attributed this discrepancy to the many different positions possible for ears, suggesting that ears' position should be examined in spatiotemporal data acquisition (49, 90). Similarly, Feighelstein et al. (100) utilized 48 facial landmarks selected based on the CatFACS and manually annotated for developing their automated model. Landmark-based approaches are by their nature better able to directly measure and thus better account for morphological variability. However, the downside of this route is the resource and effort needed for landmark annotation, given that this requires manual completion (114).

#### 4.4.2 Deep learning approach

Deep Learning approaches are gaining popularity in APR due to their reduced need for annotation and manual feature crafting. Unlike LM-based methods, DL is less sensitive to facial alignment (100), although the accuracy of the models improves with data cleaning (102). Deep learning trains artificial neural networks with many layers to automatically extract hierarchical features from vast datasets like video data. Convolutional Neural Networks (CNNs) are particularly effective for image processing tasks like classification and object recognition, offering superior performance by mapping individual inputs to single outputs. Deep learning relies heavily on large volumes of video data for training (6). Continual advancements in DL methods for APR are expanding the possibilities in the field. CNNs, inspired by the functioning of the retina, consist of various layers, including convolutional layers for feature detection, non-linearity layers to introduce non-linearity, and pooling layers for down-sampling parameters. This architecture culminates in a fully connected layer for final processing, where each node in the output layer connects directly to a node in the previous layer. Among the diverse CNN architectures, the Visual Geometry Group (VGG) 16 architecture, with its 16 convolutional layers, each equipped with 3 × 3 filters, is particularly notable for its extensive utilization in CV applications. Other advanced neural networks, such as deep residual networks (ResNets), enable the handling of deeper architectures and improved performance (95, 100, 102). These advancements in DL methods have equipped researchers and practitioners with more powerful tools for APR. It is crucial to emphasize the significance of large and diverse datasets in DL methods for APR. While DL methods are often effective, they frequently lack interpretability, which poses a challenge for humans to comprehend their decision-making process.

Building upon the work of Finka et al. (69), Feighelstein et al. (100) explored both LM-based and DL methods in APR for cats, achieving comparable accuracies of around 72%. However, DL approaches faced challenges with highly homogeneous datasets, which affected their performance. The model showed improvement when applied to a more diverse population. A similar limitation was observed by Lencioni et al. (10), who extracted 3,000 frames from seven horses of similar breed and age to classify pain following a painful stimulus and general anesthesia. Using CNN-based individual training models for each facial part, they achieved an accuracy of 90.3% for the ears, 65.5% for the eyes, and 74.5% for the mouth and nostrils, and an overall accuracy of 75.8%. This underscores the need for diverse datasets to enhance the performance of DL methods in APR. When Feighelstein et al. (102) used a DL approach for recognizing pain in 28 rabbits undergoing an orthopedic procedure, the initial “naïve” model trained on all frames achieved an accuracy of over 77%. The performance improved to over 87% when a frame selection method was applied to reduce noise in the dataset (102). Another notable DL model is the deep recurrent video model used by Broomé et al. (96, 98), which utilizes a ConvLSTM layer to analyze spatial and temporal features simultaneously, yielding better results in spatiotemporal representations. Steagall et al. (106) and Martvel et al. (114) introduced a landmark detection CNN-based model to predict facial landmark positions and pain scores based on the manually annotated FGS.

### 4.5 Limitations and downfalls in animal APR

Data imbalance is a significant challenge in both classic ML and DL methods. This issue, as highlighted by Broomé et al. (90), occurs when they are fewer instances of one class compared to another, potentially skewing the model accuracy, especially in extreme categories. In the case of animal pain recognition, there are often fewer instances of animals in pain compared to non-painful animals (97, 98). The use of data augmentation techniques, such as synthesizing additional data using 3D models and generative AI (5) has been proposed to address this imbalance. However, the highly individualized nature of pain perception and expression in animals may limit the clinical value of these techniques in animal pain recognition.

Overfitting and underfitting are frequently encountered problems in ML. Overfitting happens when a model excessively learns from the training data, resulting in inadequate performance when applied to new data. On the other hand, underfitting occurs when a model does not perform well even on the training set. Cross-validation techniques mitigate these problems by splitting the data into training, validation, and testing sets. For smaller sample sizes, ensuring that each subject appears in only one part of the data (training, validation, or testing) can be beneficial (6, 102). In DL, it is crucial to reserve a fully held-out test set comprising data from subjects not seen during training to ensure unbiased evaluation. Techniques like leave-one-subject-out cross-validation can help reduce bias by rotating subjects between the training and testing sets (96, 100). Additionally, when training DL models from scratch, the initial setup can be influenced by a random number called a “seed.” Different seeds can lead to slightly different results each time the model is trained. To ensure robustness, training and testing are often repeated with different random seeds, and the outcomes are averaged to minimize the impact of random variations. Addressing data imbalance, overfitting, and underfitting is not just a choice but a necessity for improving the accuracy and robustness of ML and DL models in applications such as animal pain recognition. It is a crucial step that cannot be overlooked.

## 5 Discussion

This narrative review aimed to offer a comprehensive journey through the progression of research on recognizing facial expressions of pain in animals. It began with the rapid advancement of grimace pain scales, moved through the refinement of FACS for various animal species, and culminated in APR. Although APR extends beyond facial cues (98, 101), existing studies' predominant focus has been analyzing pain AUs in datasets crafted through prior facial expression research and annotation.

Pessanha et al. (5) underscored several significant challenges encountered in detecting APR in animals. The first among these challenges is the scarcity of available datasets, a notable contrast to the abundance of databases in the human domain (115). Very few current datasets have been created specifically for CV and APR studies. The majority of researchers have out-sourced their dataset from previous studies (6, 84, 96, 98, 99, 101, 104, 105), with the significant advantage was that most of these dataset were already annotated for pain AUs. However, as highlighted, most of the previously published pain scales based on facial expressions were based on unspecified or artificially created ethograms and they did not undergo complete validation, except for Evangelista et al. (21). Most interestingly, they were developed before or independently from the development of the AnimalFACS dataset for the species. While agreement was often found for many AUs developed before and after AnimalFACS (62, 76), this issue may introduce inherent bias. One solution proposed for overcoming the scarcity of data is data augmentation (5, 100). However, one of the primary ethical concerns is the integrity and representativeness of the augmented data. Augmented data should accurately represent real-world variations, and care should be taken to ensure that the augmented data does not lead to misinterpretations that could result in harm or unnecessary interventions (116). To address the issue of small datasets, open access to datasets and sharing between researchers is crucial. Fairly implementing AI in veterinary care requires integrating inclusivity, openness, and trust principles in biomedical datasets by design. The concept of openly sharing multiple facets of the research process—including data, methods, and results—under terms that allow reuse, redistribution, and reproduction of all findings has given birth to open science, a practice strongly supported by several institutions and funding agencies (49, 117). Secondly, animals may have much more significant facial texture and morphology variation than humans. While initially perceived as a challenge, this may be advantageous when employing a DL approach. Finally and foremost, a significant limitation in animal APR is the need for consistent ground truth. Unlike in humans, where self-reporting of the internal affective state is commonly used, there is no verbal basis for establishing a ground truth label of pain or emotional state in animals. Consequently, animal pain detection heavily relies on third-party (human expert) interpretation, introducing intrinsic bias that cannot be bypassed entirely. One possible strategy for establishing ground truth involves designing or timing the experimental setup to induce pain. However, since pain is a subjective experience, this approach may not eliminate bias. Additionally, the type and duration of pain need to be researched further, as there are postulations about differences in facial expressions of pain between acute nociceptive and chronic pain and on the effects of general anesthesia (14, 47, 95). These hypotheses, for example, could be tested to improve the understanding and detection of pain in animals. Currently, the best way to address this problem is by using fully validated pain scales to discriminate the pain status.

In conclusion, the advancement of animal APR has immense potential for assessing and treating animal pain. However, it requires addressing data scarcity, ensuring the ethical use of augmented data, and developing consistent and validated ground truth assessments. Open science practices and collaboration will be crucial in overcoming these challenges, ultimately improving the welfare of animals in research and clinical settings.

## Author contributions

LC: Conceptualization, Data curation, Funding acquisition, Investigation, Resources, Writing – original draft, Writing – review & editing. AG: Investigation, Methodology, Writing – original draft, Writing – review & editing. GC: Investigation, Methodology, Writing – original draft, Writing – review & editing.
